# Risk Stratification in Acute Myeloid Leukemia Using CXCR Gene Signatures: A Bioinformatics Analysis

**DOI:** 10.3389/fonc.2020.584766

**Published:** 2020-10-30

**Authors:** Cong Lu, Jiang Zhu, Xiangjun Chen, Yanjie Hu, Wei Xie, Junxia Yao, Shiang Huang

**Affiliations:** ^1^ Center for Stem Cell Research and Application, Union Hospital, Tongji Medical College, Huazhong University of Science and Technology, Wuhan, China; ^2^ Institute of Hematology, Union Hospital, Tongji Medical College, Huazhong University of Science and Technology, Wuhan, China; ^3^ Biological Targeted Therapy Key Laboratory in Hubei, Wuhan, China

**Keywords:** acute myeloid leukemia, CXC chemokine receptor, FAB subtypes, risk stratification, gene signature, prognostic role

## Abstract

The role of CXC chemokine receptors in tumors has been an increasingly researched focus in recent years. However, significant prognostic values of CXCR members in acute myeloid leukemia are yet to be explored profoundly. In this study, we firstly made an analysis of the relationship of CXCR family members and AML using samples from TCGA. Our results suggested that transcriptional expressions of CXCRs serve an important role in AML. CXCR transcript expressions, except CXCR1 expression, were significantly increased in AML. It displayed the expression pattern of CXCR members in different AML subtypes according to FAB classification. The correlations of CXCR transcript expression with different genotypes and karyotypes were also present. High CXCR2 expression was found to have a significantly worse prognosis compared with that of low CXCR2 expression, and CXCR2 was also found to be an independent prognostic factor. We also established a CXCR signature to identify high-risk subgroups of patients with AML. It was an independent prognostic factor and could become a powerful method to predict the survival rate of patients.

## Introduction

Acute myelocytic leukemia (AML) is a malignant clonal disease of hematopoietic stem cells, which is characterized by a block of leukemic blasts in the bone marrow and other tissues (1). Adult patients could attain complete remission (CR) after standardized chemotherapy treatment; however, the short duration of CR is still an urgent problem for clinician due to a high relapse rate (2). Recent studies suggest that the interaction of the CXC chemokine receptor (CXCR) members and their ligands as well as the complex regulatory network of them take an effect on certain tumor-related processes (3) including activation, proliferation and invasion of leukemic cells (4).

The CXCR family consists of proteins CXCR1–7 (5). CXCR1 and CXCR2 exhibit a high affinity toward a common ligand IL-8. This receptor-ligand interaction induces leukocyte chemotaxis, cell proliferation, and migration and is critical for inflammation and metastasis of tumors (6–8). The CXC chemokine ligand (CXCL) 9, 10, 11/CXCR3 axis regulates tumor differentiation and activation and the paracrine signal transduction for immune cell development (9, 10). CXCR4, expressed on the surface of hematopoietic stem cells and leukemia blast cells, is activated by CXCL12, and it participates in leukemia cell proliferation and infiltration, as well as in conferring resistance to chemotherapy drugs (11–13). CXCL13 is the ligand for CXCR5, also known as the Burkitt lymphoma receptor 1 (BLR1), and the CXCL13/CXCR5 axis is necessary for B cell homing to lymph node follicles and for the production of immunoglobulin, which coordinates the humoral immunity of the body (14). CXCL16 selectively binds to its sole receptor, CXCR6, and is mainly expressed in natural killer, CD8^+^ T, and CD4^+^ T cells. CXCL16/CXCR6 binding plays an essential role in cell adhesion and activation of the immune response (15). CXCR7 is a receptor of CXCL12; however, it is unable to mediate G-protein activation to directly induce cell migration and is considered to be an atypical chemokine receptor (ACKR3). Ligand CXCL-11 or CXCL-12, when bound to CXCR7, can rapidly mediate ligand internalization and degradation (16).

## Materials and Methods

### Original Data

Original expression and clinical data of CXCR family members in AML were down from The Cancer Genome Atlas (TCGA) database (http://Cancegenome.nih.gov/). They were divided into eight subtypes (M0-M7) according to the French-American and British (FAB) classification of AML. The CXCR family mRNA expression level, gender, age, survival status, survival time, risk stratification, chromosome karyotype analysis, gene detection, and other clinical data were extracted from AML patients in TCGA. This study was approved by the Ethics Committee at hospital. Validation of mRNA prognostic power in this study used expression data of TARGET database.

### GEPIA

Differences of the expression levels of CXCRs between AML patients and normal tissues were obtained from the GEPIA website (http://gepia.cancer-pku.cn/). the Gene Expression Profiling Interactive Analysis server (GEPIA) is a newly developed interactive web server (17) for analyzing the RNA sequencing expression data of thousands of tumor and normal samples from the TCGA and the GTEx projects using a standard processing pipeline (18). Since AML samples from TCGA database were all tumor samples, GEPIA matches normal samples from the GTEs database.

### cBioPortal

cBioPortal (http://www.cbioportal.org) is a multi-functional open network platform, which is a set of tools that propose data mining, data integration, and visualization based on the TCGA database. The intuitive web interface enables the complex cancer genome profiles to be integrated and explored by clinicians (19).

### STRING

STRING (https://string-db.org/) is a database of known and predicted protein-protein interactions networks designed for protein functional enrichment analysis. The interactions include direct (physical) and indirect (functional) associations. They stem from computational prediction, from knowledge transfer between organisms, and from interactions aggregated from other (primary) databases. The STRING database currently covers 24,584,628 proteins from 5,090 organisms (20, 21). The Search Tool by choosing Multiple Proteins was used to construct the PPI networks of CXCR member

### Statistical Methods

SPSS 19.0 software (SPSS, Chicago, IL) and R language (3.6.3) were used for the statistical analysis. Non-parametric tests were used to compare two or more independent sample sets of data. The patient samples were divided into high- and low- expression groups based on their median expression value. Kaplan-Meier survival analysis and log-rank tests were conducted to analyze the overall survival (OS) using survival R package. Univariate and multivariate analyses were performed on the categorized data to identify independent predictors of outcome. *P* values below 0.05 were considered significant. We chose the candidate genes and they were subjected to multiple proportional risk regression to construct a gene signature as a risk score model. The risk score model included the expression level of mRNA for each optimal prognosis, with weights determined by the estimated regression coefficients of their multivariate Cox regression model, as shown below

$$\text{Risk Score}\;(\mathrm{patient})=\sum_{\text{i}}\;\text{Coefficient}\;(\text{mRNAi})*\text{expression}\;(\text{mRNAi})$$

## Results

### Transcript Expression of *CXCRs* in Patients With AML

**Figure 1:**
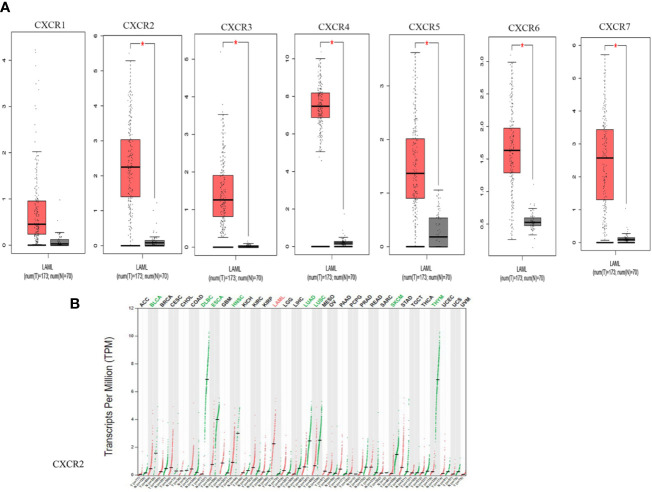
The mRNA expression from GEPIA. **(A)** CXCRs between AML and normal samples. (*P < 0.05). **(B)** CXCR2 expression profile across all tumor samples and paired normal tissues.

We compared the transcript expressions of the *CXCRs* in patients with AML with those of normal control subjects, using server GEPIA (22). 173 patients with AML from TCGA and 70 normal subjects from the Genotype-Tissue Expression (GTEx) portal were displayed on the website. Remarkably, all *CXCR* transcripts, except for *CXCR1*, showed a higher expression of mRNA in patients with AML, in comparison to that in normal subjects (**Figure 1A**). Of note, the expression of the *CXCR4* transcript was much higher in the AML group. The decreasing order of expression levels was as follows: *CXCR4*, *CXCR7*, *CXCR2*, *CXCR6*, *CXCR5*, *CXCR3*, and *CXCR1*. Although, *CXCR2* expression is down-regulated in samples from most types of cancers compared to samples from normal subjects, it was found to be upregulated in AML (**Figure 1B**).

### Transcript Expression of *CXCRs* in French-American and British (FAB) Subtypes of AML

**Figure 2:**
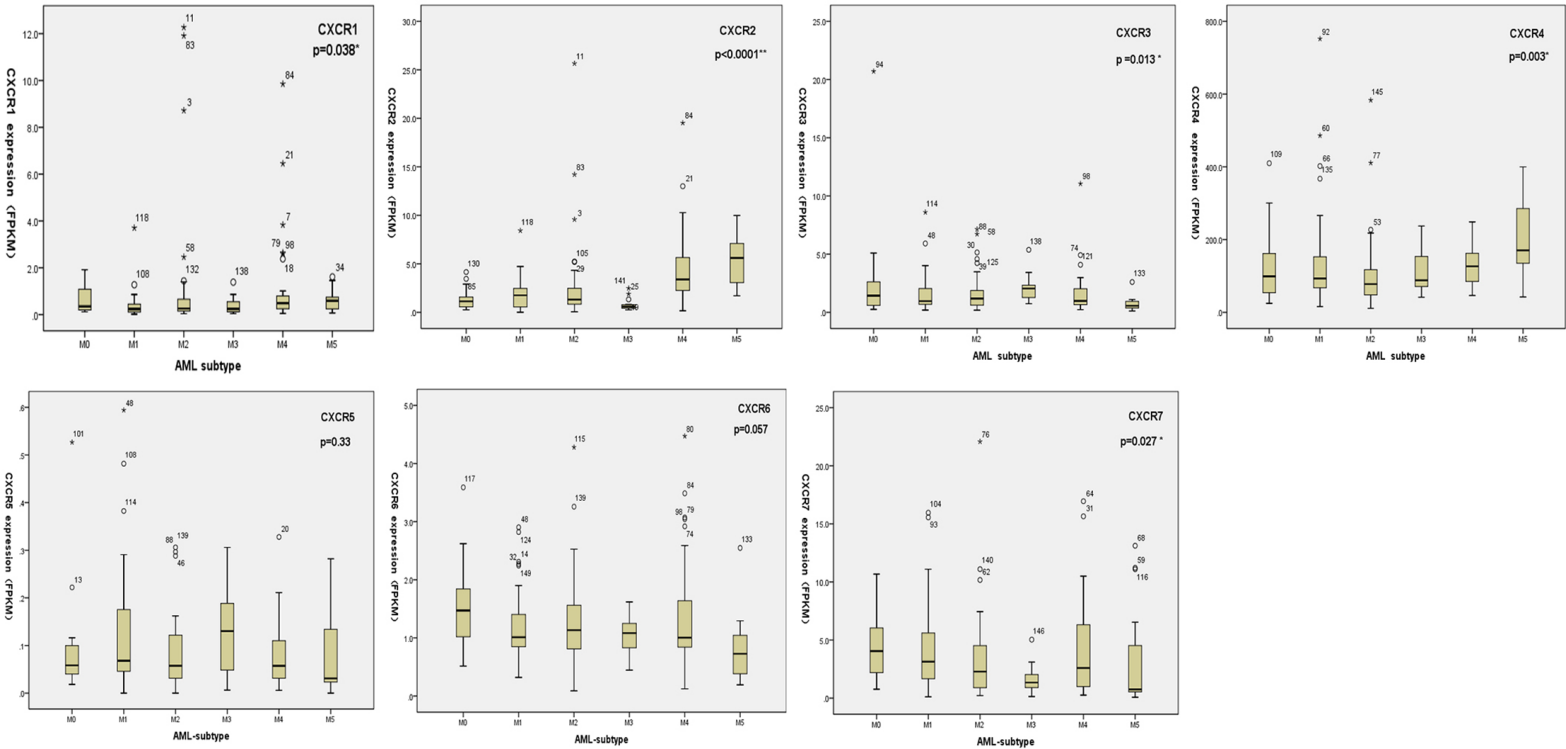
The transcriptional expressions of CXCRs in AML subtype according to FAB classification.

We analyzed transcript expression of each *CXCR* in the M0 to M5 FAB subtypes of 151 patients with AML; two cases of AML-M6 and one case of AML-M7 were not analyzed. Higher expression of *CXCR1* transcripts was found more frequently in the AML-M5 subtype than in other subtypes (*P* = 0.0038). *CXCR2* showed the highest expression in AML-M5, and the lowest expression was seen in AML-M3 (*P* < 0.0001). *CXCR3* transcripts showed a higher expression in AML-M3, as compared to that in the other subtypes (*P* = 0.013). AML-M4/M5 patients showed an increased expression of *CXCR4* transcripts compared to that in other subtypes, with the highest expression found in AML-M5 (*P* = 0.003). Moreover, no significant difference was found between the expressions of *CXCR5* and *CXCR6* among the different AML subtypes. *CXCR7* showed the highest expression in AML-M0, while the lowest expression was observed in AML-M5 patients (*P* = 0.027) (**Figure 2**).

### Correlation of *CXCR* Transcript Expression With Different Genotypes and Karyotypes

**Figure 3:**
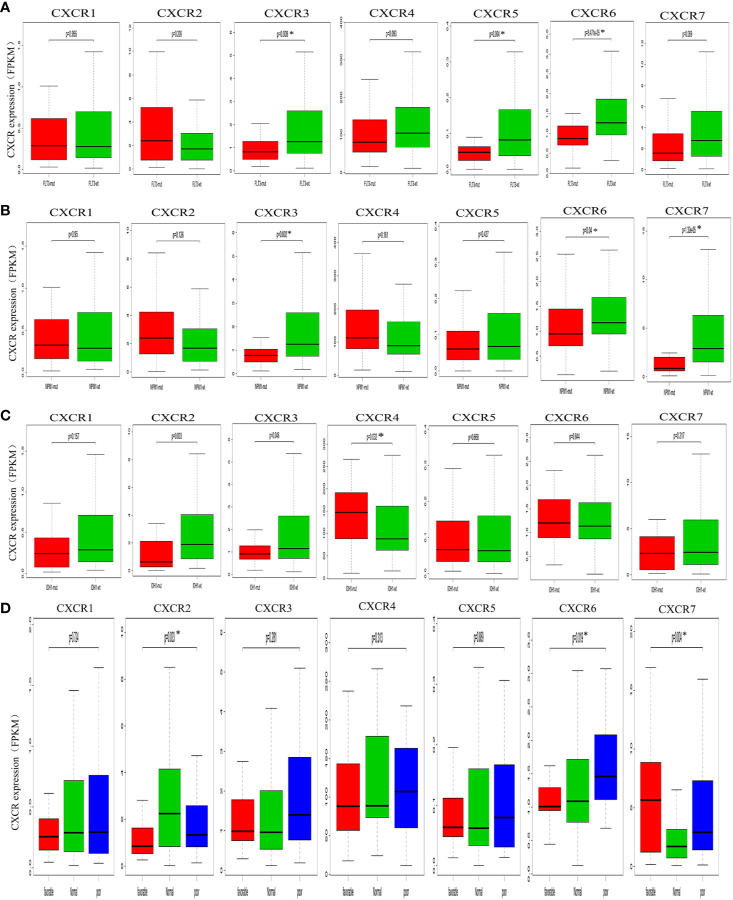
Analysis of prognostic gene expressions between the mutant group and the wild-type group. **(A)** *FLT3* mutation. **(B)** *NPM1* mutation. **(C)** *IDH1* mutation. **(D)** cytogenetic karyotype.

To assess the relationship between *CXCR* transcript expression and the mutation status of traditional prognostic genes, we compared transcript expression of *CXCRs* in patients having mutations in *FLT3*, *IDH1*, and *NPM1*, with that in the mutation-negative control group. Patients with a *FLT3* mutation showed a significant decrease in the expression of *CXCR3*, *CXCR5*, and *CXCR6* transcripts compared to that in mutation-negative patients (**Figure 3A**). Similarly, patients with a *NPM1* mutation showed a significant decrease in the expression *CXCR3* and *CXCR6* transcripts compared to that in mutation-negative patients (**Figure 3B**). As for the mutant *IDH1*, the *CXCR4* transcript showed a significant increase in its expression in mutant *IDH1* patients compared to that in the control group (P = 0.032, **Figure 3C**)

We also analyzed the relationship between the *CXCR* transcript expression and cytogenetic karyotype, which is an important parameter for AML prognosis. Patients with the karyocyte t(15;17), t(8;21), and inv(16) were designated the favorable group; patients with normal karyocytes were designated the normal group; patients with the karyocyte 5q-/7q- or complex karyotype were designated the poor group. The results showed that patients of the favorable group had the highest *CXCR7* expression (P = 0.004), those of the normal group had the highest *CXCR2* transcript expression (P = 0.023), and those of the poor group had the highest *CXCR6* transcript expression (P = 0.019, **Figure 3D**).

### Correlation of *CXCR* Transcript Expression With AML Risk Stratification

**Figure 4:**
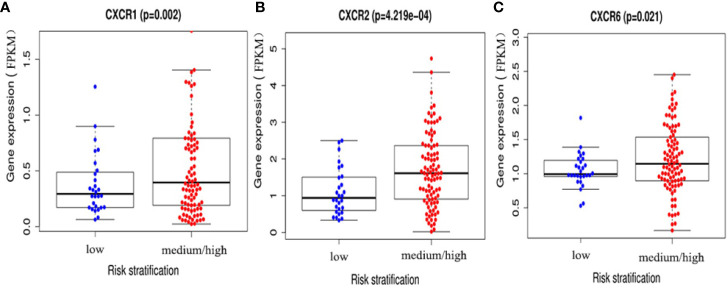
The expressions of **(A)** CXCR1, **(B)** CXCR2, and **(C)** CXCR6 between AML low and medium-high risk group.

To assess the diagnostic and prognostic significance of *CXCRs* in AML, the correlation between expression of each *CXCR* transcript with AML risk stratification was estimated. The patients were divided into two groups according to their risk stratification. The intermediate-risk and high-risk patients were combined into one medium/high-risk group, and the expression of each *CXCR* transcript in the medium/high-risk group was compared to that of the low-risk group. The expression of *CXCR1*, *CXCR2*, and *CXCR6* transcripts was significantly higher in patients of the medium/high-risk group than that in patients of the low risk group (*P* < 0.05) (**Figure 4**).

### Clinical Characteristics of 122 TCGA Samples of Patients With AML

The information of 122 patients with AML who are eligible for survival analysis was retrieved from TCGA database. The screening criteria for these patients as follows: 1) complete survival data present; 2) survival time longer than 30 days; 3) RNA-sequencing expression data present. The relevant clinical characteristics are shown in **Table 1**.

**Table 1:** Clinical characteristics of 122 AML patients enrolled from the TCGA database for survival analysis.

Characteristic	Category	Cases
Age	<60 years	77
	>= 60 years	45
Gender	Male	66
	Female	56
FAB-subtype	M0	12
	M1	28
	M2	29
	M3	12
	M4	26
	M5	12
	M6	2
	M7	1
karyotype	Normal/mediate	65
	Poor	22
	Favorable	24
	NA	11
FLT3 gene	Mutant	34
	WT	84
	NA	4
NPM1 gene	Mutant	29
	WT	89
	NA	4
IDH1 gene Survival state	Mutant WT Alive Dead	22 100 51 71

### The Role of *CXCRs* in AML Prognosis

**Figure 5:**
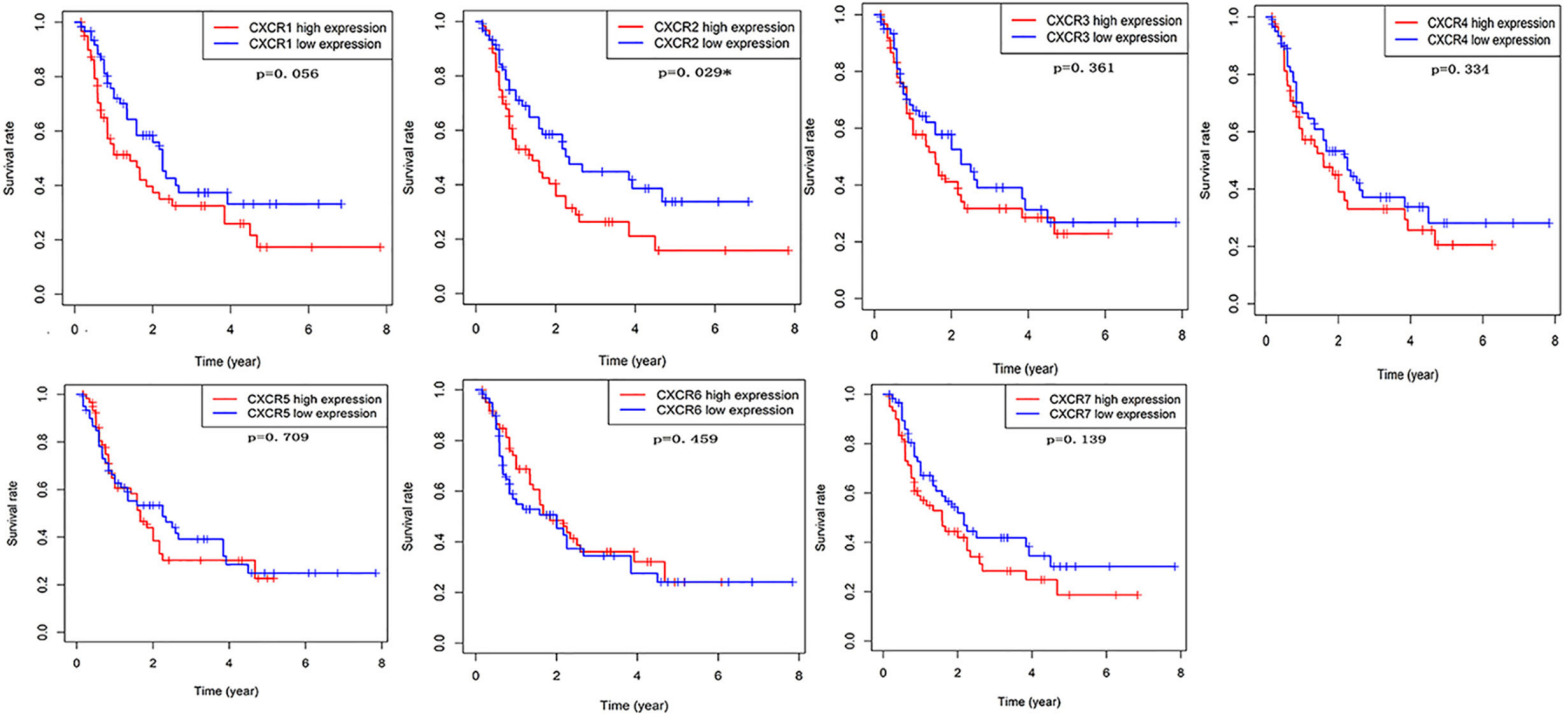
The prognostic values of CXCR family members in AML patients.

The prognostic value of *CXCRs* was estimated by analyzing the survival data and *CXCR* transcript levels in 122 patients from TCGA database. For each *CXCR* transcript, patients were divided into either the high- or low-expression group according to the median value of *CXCR* transcript expression. The median values of each CXCR mRNA expression (FPKM) were shown as follows: CXCR1 0.287, CXCR2 1.772, CXCR3 1.052, CXCR4 92.207, CXCR5 0.065, CXCR6 1.137, and CXCR7 2.400. Using Kaplan-Meier survival analysis, it was found that the survival time of the *CXCR2* high-expression group was significantly shorter than that of the low-expression group (*P* = 0.029). However, no difference in survival times was observed between the high- and low-expression groups for other *CXCR* transcripts (**Figure 5**).

Furthermore, univariate and multivariate COX analyses were used to determine whether *CXCR2* was an independent prognostic factor. Gender, age, white blood cell (WBC) counts, blast cell percentage, risk stratification, and *FLT3* and *IDH1* gene mutation were also analyzed to identify the independent factors affecting patient survival. Age, risk stratification, and *CXCR2* expression were found to be the independent factors affecting patient survival (**Table 2**).

**Table 2:** Univariate and multivariate overall survival (OS) analysis of CXCR2 in AML patients.

parameter	Univariate analysis			Multivariate analysis		
	HR	95% CI	*P* value	HR	95% CI	P value
Gender(male/female)	0.937	0.586–1.497	0.785	1.183	0.705–1.987	0.522
Age(<60 y/>=60y)	2.268	1.415–3.636	0.00066**	2.153	1.264–3.665	0.0047*
WBC(<2G/L/>=2G/L)	1.245	0.779–1.991	0.359	1.028	0.579–1.824	0.924
Blast (%)	1.005	0.996–1.015	0.261	1.004	0.994–1.015	0.390
Risk stratification (low/median/high)	1.822	1.274–2.606	0.0010*	1.540	1.029–2.305	0.036*
*FLT3* (mut/wt)	1.392	0.840–2.305	0.199	1.286	0.719–2.297	0.396
*IDH1*(mut/wt)	0.832	0.446–1.549	0.561	0.771	0.392–1.517	0.451
CXCR2 (high/low)	1.226	1.067–1.408	0.0039*	1.185	1.005–1.398	0.043*

*p > 0.001, **P < 0.001 (P < 0.05)

### Prognostic Value of *CXCR* Signature in AML

Given the increasing focus on the prognostic value of gene signatures, and the prognostic significance of *CXCR* transcripts in AML, the potential of *CXCR* signatures as a risk score model for AML was explored.

We chose *CXCR1–7* as the candidate genes; these were subjected to multiple proportional risk regression analysis to construct a risk score model based on gene signatures. The risk score model was constructed as follows:

Risk score=0.65× (CXCR1exp.)+2.01×(CXCR2exp.)+1.89× (CXCR3exp.)+0.65× (CXCR6exp.)

where exp. represents expression levels.

**Figure 6:**
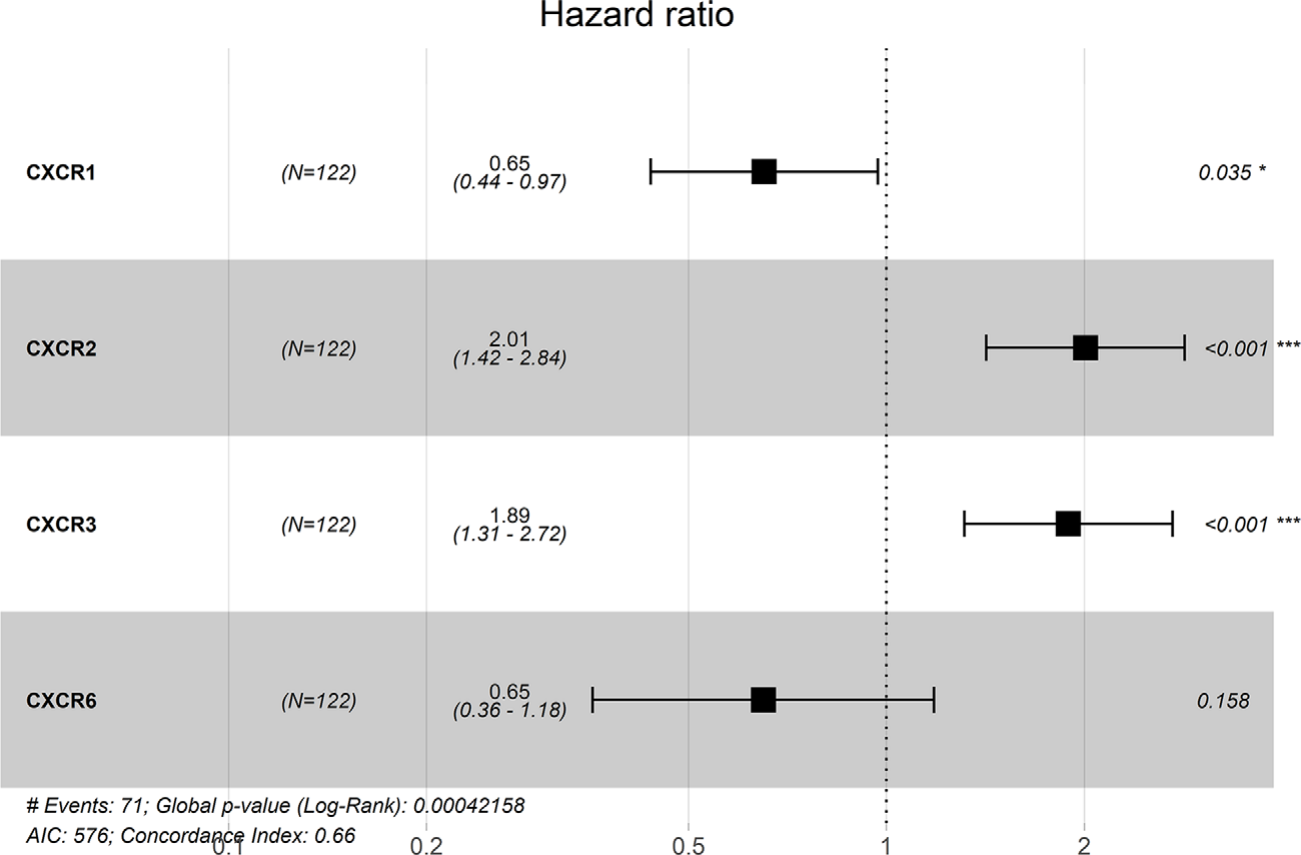
The forest plot for the gene signature model and Concordance Index.

**Figure 7:**
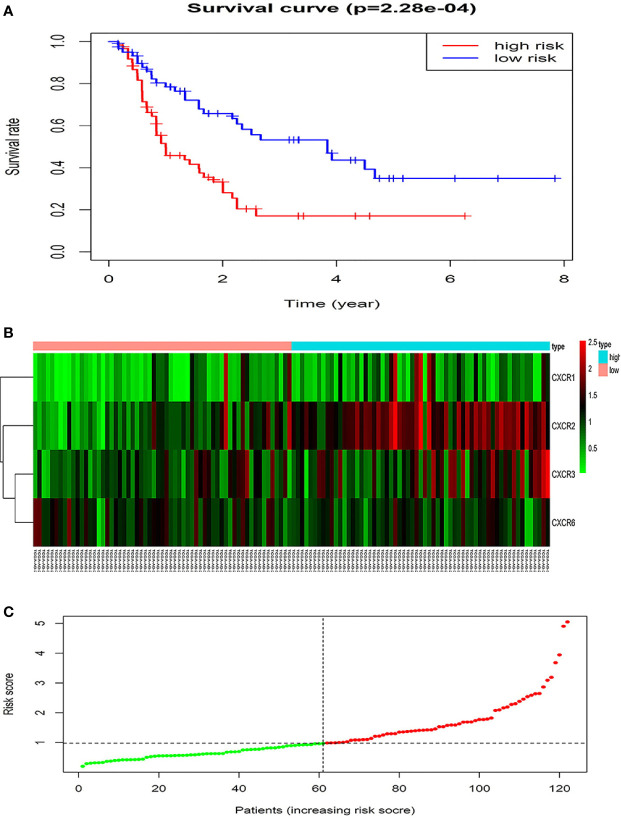
The prognostic values of CXCR signature. **(A)** The survival curves of low- and high- risk group. **(B)** The heatmap of gene signature. **(C)** The risk score of AML patients in two groups.

**Figure 8:**
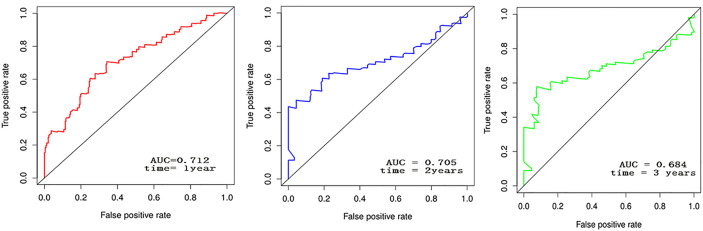
The AUC corresponding to 1, 2, and 3 years of CXCR signature using K-M plot.

The forest plot for the gene signature model and its concordance index (C-index = 0.66, *P* = 4.216e-4) are shown in **Figure 6**. The patients were divided into high- and low-risk groups according to the median risk score, and it was found that the high-risk group displayed a significant reduction in OS compared to that of the low-risk group (*P* = 2.28e−04, **Figure 7A**). The heatmap of the core genes and risk-score of patients with AML in the two groups are shown in **Figures 7B, C**. Additionally, receiver operating characteristic (ROC) curves were built to evaluate the performance of the *CXCR* signature risk model at three time points (**Figure 8**). The AUC corresponding to 1, 2, and 3 years was 0.719, 0.705, and 0.684, using the Kaplan-Meier method.

To determine if *CXCR* signature risk score could be used as an independent prognostic predictor for OS, we performed univariate and multivariate Cox regression analysis. The factors included were similar to those used in the previous regression analysis. The results showed that *CXCR* signature risk-score was an independent prognostic predictor of OS. Additionally, we found that age greater than 60 years and high-risk of AML (in the traditional risk stratification) were also independent predictors of a shortened OS (**Table 3**).

**Table 3:** Univariate and multivariate overall survival (OS) analysis of CXCR signature in AML patients.

parameter	univariate analysis			multivariate analysis		
	HR	95%CI	*P* value	HR	95%CI	*P* value
Gender(male/female)	0.950	0.594–1.518	0.830	1.148	0.683–1.930	0.602
Age(<60y/>=60y)	2.240	1.397–3.592	0.00081**	1.882	1.086–2.261	0.024*
WBC(<2G/L/>=2G/L)	1.262	0.789–2.017	0.331	1.065	0.622–1.825	0.817
Blast (%)	1.005	0.996–1.015	0.245	1.005	0.994–1.015	0.382
Risk stratification (low/median/high)	1.856	1.296–2.659	0.00073**	1.551	1.012–2.374	0.043*
*FLT3* (mut/wt)	1.416	0.855–2.346	0.176	1.434	0.797–2.576	0.228
*IDH1*(mut/wt)	0.861	0.462–1.604	0.637	0.949	0.485–1.858	0.879
Riskscore (high/low)	2.041	1.626–2.564	8.23E-10**	1.944	1.516–2.493	1.62E-07**

*p > 0.001, **P < 0.001 (P<0.05).

### Mutation Status of *CXCRs* and Their Correlation With Each Other

We used the c-Bioportal online tool to estimate the correlation between the *CXCRs* (RNA Seq V2 RSEM); the Pearson’s correlations are listed in **Table 4**. The data showed that *CXCR1* expression was associated with *CXCR2* (r = 0.523). The expression of *CXCR3* was found to be closely correlated to that of *CXCR5* (r = 0.538) and *CXCR6* (r = 0.412).

**Table 4:** The correlations of CXCRs with each other in AML (RNA Seq V2 RSEM).

	CXCR1	CXCR2	CXCR3	CXCR4	CXCR5	CXCR6	CXCR7
CXCR1	1	0.523*	**0.258**	0.0974	**0.274**	0.0902	−0.0495
CXCR2	0.523*	1	−0.0566	**0.142**	3.61E-03	−0.0548	−0.0678
CXCR3	**0.258**	−0.0566	1	−0.025	0.538*	0.412*	**0.199**
CXCR4	0.0974	**0.142**	−0.052	1	0.0838	−0.0397	−0.159
CXCR5	**0.274**	3.61E-03	0.538*	0.0838	**1**	**0.367**	−0.0532
CXCR6	0.0902	−0.0548	0.412*	−0.0397	**0.367**	1	0.155
CXCR7	−0.0495	−0.0678	**0.199**	−0.159	−0.0532	0.155	1

Red indicates positive correlation, blue indicates negative correlation, and the depth of color indicates the degree of correlation.

### Protein-Protein Interaction Network and Functions of *CXCR* and Their Neighboring Genes

**Figure 9:**
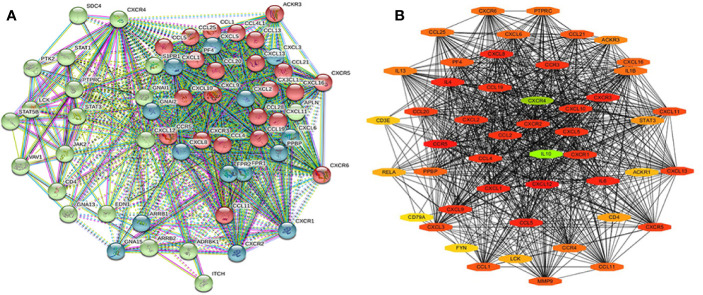
The PPI of CXCR family members and their neighbor genes **(A)** the PPI network analyzed by String. **(B)** The hub genes analyzed by Cytoscape.

The STRING database was used to cluster and construct a network of *CXCRs* and the 47 most frequently altered neighboring genes (**Figure 9A**). Cytoscape software was used to screen the hub genes from the constructed network. *CXCR4* and *IL-10* were found to be important hub genes of the network (**Figure 9B**).

**Figure 10:**
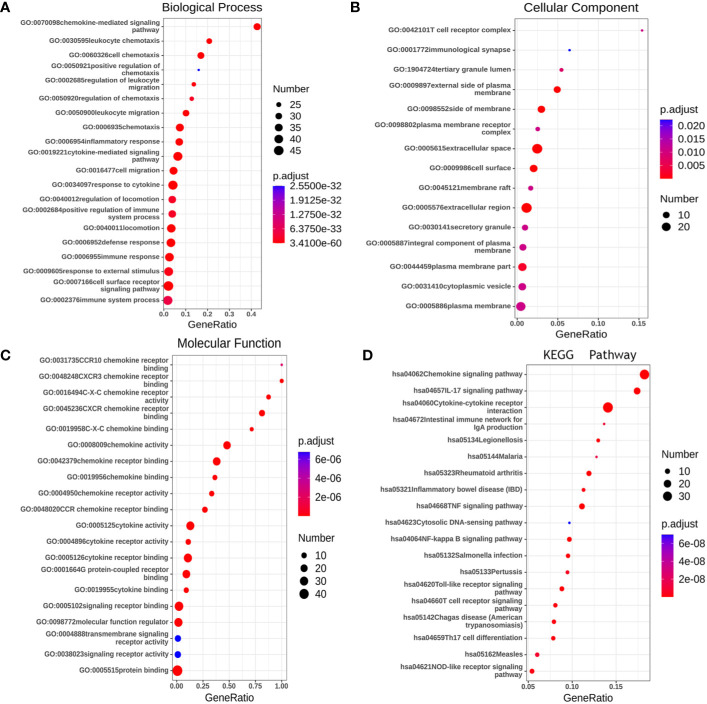
The functions of CXCRs and neighborhood genes predicted by GO and KEGG analysis by String. **(A)** Biological Process. **(B)** Cellular Component. **(C)** Molecular Function. **(D)** KEGG enrichment.

Gene ontology (GO) enrichment analysis was used to predict the functional role of target host genes, and classify them into functional categories, including biological process (BP), cellular component (CC), and molecular function (MF). GO:0070098 (chemokine-mediated signaling pathway), GO:0030595 (leukocyte chemotaxis), GO:0060326 (cell chemotaxis), GO:0019221 (cytokine-mediated signaling pathway), and GO:0007166 (cell surface receptor signal pathway) were found to be significantly regulated by *CXCRs* and were classified under BP (**Figure 10A**) (23). These are well-known signal pathways involving cytokines and their receptors. Several important GO enriched BP pathways are associated with anti-inflammatory and immune responses. The top 20 GO terms for CCs and MF are shown in **Figures 10B, C**.

We used the Kyoto Encyclopedia of Genes and Genomes (KEGG) to functionally analyze the *CXCRs*, as well as frequently altered neighboring genes. The 20 most significantly *CXCR*-enriched pathways, using KEGG analysis in patients with AML, are shown in **Figure 10D** (P < 0.0001). KEGG pathway analysis showed that these *CXCR* genes are most significantly enriched in cytokine-cytokine receptor interaction, chemokine signaling pathway, IL-17 signaling pathway, TNF signaling pathway and NF-kappa B signaling, and Toll-like receptor signaling pathway, which is related to the process of tumor invasion and metastasis.

### Validation in the TARGET Database

**Figure 11:**
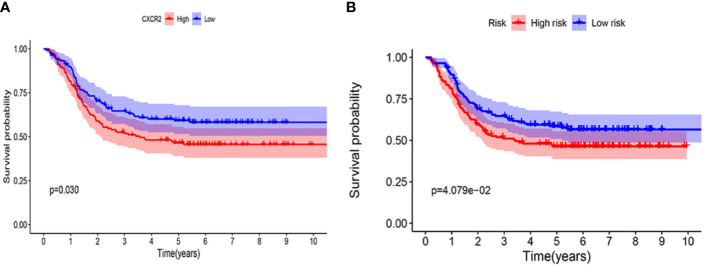
Kaplan-Meier survival curves in validation cohort using TARGET database. **(A)** CXCR2. **(B)** CXCR gene signature.

The prognostic value of *CXCR2* and the *CXCR* signature risk score model were verified using the TARGET database. We selected 295 patients with AML from the TARGET database to analyze the prognostic value of *CXCR* genes and evaluate the risk score model. The median value of CXCR2 expression (counts) in this cohort was 65. It was observed that *CXCR2* could act as an independent prognostic factor (P = 0.030, **Figure 11A**). We used the risk score model [Risk score = 0.65×(*CXCR1*exp.) + 2.01 × (*CXCR2*exp.) + 1.89 × (*CXCR3*exp.) + 0.65 × (*CXCR6*exp.)] established to calculate the risk score of each patient. The median value of CXCR signature risk score was 940.872. The risk score model could accurately categorize the patients in the TARGET database into high- and low-risk groups according to the median value (P = 4.079e-02, **Figure 11B**).

## Discussion

The *CXCR* family members have been reported to play important roles in different types of cancers (24, 25). Although *CXCRs* are known to be involved in tumorigenesis and prognosis of numerous cancers, there is a lack of detailed bioinformatic analyses of *CXCRs* in AML. This is the first study to investigate the transcript levels and prognostic value of *CXCRs* in AML. Additionally, we evaluated *CXCR* signatures as predictors of the risk of developing AML.

Our analysis revealed that all *CXCRs*, except *CXCR1*, were up-regulated in patients with AML compared to normal subjects. Notably, *CXCR2* expression was found to significantly increase in AML but is known to be no apparent increase or even decreased in most of other tumors, indicating a critical role of *CXCR2* in AML.

We also showed that *CXCR4* expression was increased AML-M4 and AML-M5. This is consistent with the findings of our previous study, wherein we demonstrated that *CXCR4* expression is higher in AML-M4 and AML-M5 than in subtypes M2 and M3 (26). Further, we found that the transcript expression of other *CXCRs* was significantly different in different AML subtypes. Very few studies have reported the correlation between the FAB subtypes and transcript levels of *CXCRs*; however, this correlation needs to be investigated further.

We assessed the correlation between *CXCR* transcript expression and the cytogenetic and molecular profile of patients with AML. Previous studies have reported a correlation between *CXCR4* and *FLT3* in AML. Rombouts et al. showed that *CXCR4* showed a higher expression in *FLT3*-ITD mutant group than in the *FLT3*-wild-type group (27). This finding is consistent with our previous study as well (26). These results showed that patients with a *FLT3* mutation had lower expression of *CXCR3*, *CXCR5*, and *CXCR6* transcripts than that in mutation-negative patients, suggesting a possible anti-cancer effect of *CXCR3/5/6* in AML. However, we have not been able to demonstrate this.

Konoplev et al. evaluated the relationship between *CXCR4* and *NPM1* in a group of 117 untreated adults with AML and found that a mutation in NPM1 is not correlated with CXCR4 or pCXCR4 protein levels, suggesting that *CXCR4* and *NPM* pathways play independent roles in adult AML (28). In contrast, Mannelli et al. demonstrated that the *NPM1*-mutated cases of AML displayed a significantly higher expression of *CXCR4* compared to *NPM1*-wild-type cases (29). With respect to studies investigating the relationship between *CXCR4* and other prognostic genes, Kuo et al. found that *CXCR4* expression was significantly higher in *CEBPA* wild-type patients than in *CEBPA* mutant patients; thus, *CEBPA* has been speculated to affect the *CXCR4* expression (30). *In vitro* studies first detected the increased expression of endogenous *CXCR4* in AML cell lines and demonstrated that *CEBPA* mutants modulated CXCR4 activation. Currently, the correlation between *CXCR4/CXCR7* expression and *IDH1* has been reported in human glioma (31, 32) but has not been reported in AML yet. In this study, we found that *CXCR4* transcript expression in mutant *IDH1* patients was significantly higher than that in *IDH1* wild-type patients. This helps to better understand the relationship between *CXCR4* and *IDH1* in AML. Despite these results, there are very few reports on the correlation between other *CXCRs* and AML prognostic genes; hence, this further investigation is required.

Risk stratification for AML the molecular and genetic characteristics takes into account, as well as age, WBC count, and several other factors. Correlation analysis of *CXCRs* with AML risk stratification revealed that *CXCR1*, *CXCR2*, and *CXCR6* transcript expressions were higher in the medium/high-risk group than those in the low-risk group. To determine the prognostic value of *CXCRs*, we analyzed the OS of patients with AML. OS analysis showed that *CXCR2* was a predictor of shorter OS and was independent of other classical factors such as age, gender, WBC counts, blast cell percentage, risk stratification, and *FLT3*/*IDH1* gene mutation.

The CXCR1/CXCR2 pathway is the most widely studied pathway in tumors. CXCL1, 2, 3, and 8 are angiogenic chemokines that bind to receptor CXCR2, with the highest affinity exhibited by CXCL1 (33). While studying the effects and ligands of CXCR1/CXCR2, Cheng et al. found that CXCL8 is up-regulated in co-cultures of bone marrow mesenchymal cells and leukemia cell lines compared with CXCL8 expression in single cultures (34). Inhibition of CXCL8/CXCR2 binding can lead to cell cycle arrest in G0/G1 phase, inhibition of AML cell proliferation, inhibition of AKT phosphorylation, and cell apoptosis. Elevated expression of *IL-8* and *CXCR2* was found in stem and progenitor cells isolated from AML and myelodysplastic syndrome (MDS) patients. Schinke et al. demonstrated that an increased *CXCR2* expression was a poor prognostic factor for AML and MDS, further reinforcing the prominent role of the IL-8/CXCR2 axis in AML and MDS (35). Hao et al. investigated the relationship between CXCL1/CXCL2, clinical characteristics, and prognosis in patients with AML (36). Expression of *CXCL1* and *CXCL2* was detected using quantitative-PCR in bone marrow samples from 160 patients with *de novo* AML. Furthermore, CR was assessed and event-free survival (EFS) and overall survival were calculated. An increased expression of *CXCL2* was found to correlate with the monochromosomal karyotype (P = 0.001). However, *CXCL2* was negatively correlated with EFS (P = 0.069) and overall survival (P = 0.055), although this was not statistically significant.

The role of CXCR4 in the development of AML has become an attractive subject of investigation in the recent years. CXCL12 binds and activates its homologous receptor CXCR4 in the microenvironment of the bone marrow to mediate the transport of leukemia cells, while keeping in close contact with stromal cells and the extracellular matrix to generate growth-promoting and anti-apoptotic signals. Increased *CXCR4* expression in AML cells is associated with poor prognosis (37). Rombouts et al. found that patients with increased *CXCR4* expression in the CD34^+^ subset of cells had significantly reduced chances of survival and a higher probability of relapse, suggesting that the Stromal Cell-Derived Factor-1(SDF-1)/CXCR4 axis may influence responsiveness to therapy and contribute to an unfavorable prognosis of AML (27). Additionally, we have previously suggested that *CXCR4* is an independent prognostic factor for AML (26). The results of a previous report suggested that *CXCR4* is expressed in a subset of patients with AML and is associated with poor prognosis, and *CXCR4* expression appears to be an independent prognostic factor for reduced survival in a heterogeneous group of patients with AML (38). However, we did not find any obvious prognostic value of *CXCR4*. This may be due to insufficient samples in the dataset or erroneous methods of detection for *CXCR4* expression.

CXCR7 is a newly discovered receptor of CXCL12 that co-exists with CXCR4, and CXCR4/CXCR7 has been declared to play a role in AML (39). Kim et al. investigated the expression levels and function of *CXCR7* in AML cells *in vitro*, and showed that *CXCR7* was involved in the regulation of autocrine CXCL12 in AML cells (40). Faaij et al. analyzed the expression of chemokine receptors in children with skin involvement of AML and showed that skin-residing AML cells intracellularly expressed CXCR4 and CXCR7 in 90.9% of evaluated cases (41). These results suggested that chemokine receptor interactions are involved in the homing and retention of AML blast cells in the skin.

The role of the CXCR3, CXCR5, and CXCR6 axes in AML is not well known. The therapeutic potential of CXCR3-CXCL9/10/11 and CXCR5/CXCL13 signaling pathways in tumors has been investigated previously (42, 43). Abnormally high levels of CXCR5 and CXCL13 in the serum of lymphoma patients are significantly associated with poor prognosis (44). Similarly, abnormally high expression levels of *CXCR6* and *CXCL16* are found to be closely related to tumor proliferation and metastasis, and have been reported to be associated with human ovarian cancer (45) and the metastasis of liver cancer cells (46).

The traditional approach of predicting the prognosis of a disease using one single gene cannot compete with the predictive value of several different potential biomarkers; thus, there is a growing concern over the prognostic value of gene signatures. Yu et al. had reported a *CXCR* signature for gastric cancer, and a Kaplan-Meier survival analysis revealed that the OS significantly reduced in the high-risk *CXCR* signature group compared with that of the low-risk *CXCR* signature group (47). In this study, we established a *CXCR* signature for AML, which comprehensively determined the patient’s prognosis based on the expression of each *CXCR* transcript and its prognosis coefficient. Using the Kaplan-Meier survival analysis for the *CXCR* signature, median OS in the low risk-group was shown to be significantly higher than that of the high-risk group. Additionally, it is known AML prognosis increasingly relies on detection of multigene Panel Testing. We did a bioinformatics analysis and verified it in different database. Experimental validation need to be further studied.

In conclusion, we analyzed the expression, clinical features, and prognostic value of *CXCRs* in AML. Our results suggested that transcriptional expressions of *CXCRs* serve an important role in AML. *CXCR* transcript expressions, except *CXCR1* expression, were significantly increased in AML. High *CXCR2* expression was found to have a significantly worse prognosis compared with that of low *CXCR2* expression, and *CXCR2* was also found to be an independent prognostic factor. We established a *CXCR* signature to identify high-risk subgroups of patients with AML. These results might help improve the treatment and prognosis of patients with AML.

## Data Availability Statement

Publicly available datasets were analyzed in this study. This data can be found here: TCGA database (http://cancergenome.nih.gov/).

## Ethics Statement

The studies involving human participants were reviewed and approved by the Academic Committee of Wuhan Union Hospital, Huazhong University of Science and technology. Written informed consent for participation was not required for this study in accordance with the national legislation and the institutional requirements.

## Author Contributions

The concept of the study was designed by CL, SH, and JY. WX, YH, and CL made the literature research. JZ provided data acquisition. XC and CL made the statistical analysis. CL and JY wrote the manuscript with inputs from all co-authors. The first version was prepared by CL. WX, YH, and JZ made a revision. JY and SH conceived and coordinated the project. SH was the guarantor of integrity of entire study. All authors contributed to the article and approved the submitted version.

## Funding

This study was funded by Hubei Provincial Natural Science Foundation of China (grant no. 2018CFB485) and National Natural Science Foundation of China (grant no. 81200348)

## Conflict of Interest

The authors declare that the research was conducted in the absence of any commercial or financial relationships that could be construed as a potential conflict of interest.
